# Preclinical study of peripheral nerve regeneration using nerve guidance conduits based on polyhydroxyalkanaotes

**DOI:** 10.1002/btm2.10223

**Published:** 2021-05-21

**Authors:** Lorena R. Lizarraga‐Valderrama, Giulia Ronchi, Rinat Nigmatullin, Federica Fregnan, Pooja Basnett, Alexandra Paxinou, Stefano Geuna, Ipsita Roy

**Affiliations:** ^1^ School of Life Sciences, College of Liberal Arts and Sciences University of Westminster London UK; ^2^ School of Life Sciences, Queen's Medical Centre University of Nottingham Nottingham UK; ^3^ Department of Clinical and Biological Sciences University of Turin Turin Italy; ^4^ Neuroscience Institute of the Cavalieri Ottolenghi Foundation (NICO) University of Turin Turin Italy; ^5^ Bristol Composites Institute (ACCIS) University of Bristol Bristol UK; ^6^ Department of Materials Science and Engineering, Faculty of Engineering University of Sheffield Sheffield UK

**Keywords:** nerve regeneration, nerve tissue engineering, peripheral nerve regeneration

## Abstract

Nerve guidance conduits (NGCs) are used as an alternative to the “gold standard” nerve autografting, preventing the need for surgical intervention required to harvest autologous nerves. However, the regeneration outcomes achieved with the current NGCs are only comparable with autografting when the gap is short (less than 10 mm). In the present study, we have developed NGCs made from a blend of polyhydroxyalkanoates, a family of natural resorbable polymers. Hollow NGCs made from a 75:25 poly(3‐hydroxyoctanoate)/poly(3‐hydroxybutyrate) blend (PHA‐NGCs) were manufactured using dip‐molding. These PHA‐NGCs showed appropriate flexibility for peripheral nerve regeneration. In vitro cell studies performed using RT4‐D6P2T rat Schwann cell line confirmed that the material is capable of sustaining cell proliferation and adhesion. PHA‐NGCs were then implanted in vivo to repair 10 mm gaps of the median nerve of female Wistar rats for 12 weeks. Functional evaluation of the regenerated nerve using the grasping test showed that PHA‐NGCs displayed similar motor recovery as the autograft, starting from week 7. Additionally, nerve cross‐sectional area, density and number of myelinated cells, as well as axon diameter, fiber diameter, myelin thickness and g‐ratio obtained using the PHA‐NGCs were found comparable to an autograft. This preclinical data confirmed that the PHA‐NGCs are indeed highly promising candidates for peripheral nerve regeneration.

## 1 INTRODUCTION

Peripheral nerve injuries (PNI) may have a dramatic impact on the patient's quality of life and can involve high health care expenses. The annual incidence of PNIs in developed countries range from 13 to 23 out of 100,000 people.^1^ In Europe, a PNI incidence rate of 3.3% and 1.8% has been reported in trauma patients, for upper and lower extremity nerves, respectively. PNIs in the upper extremity are caused by bone fractures located in the humerus (37.2%) or ulna (20.3%), affecting most frequently the radial, median, ulnar and auxiliary nerves.^2^ Peroneal (51%) and sciatic nerves (25%) are the most commonly affected nerves of lower extremity trauma.^3^ Despite the tremendous advances in surgical techniques and tissue engineering, the prognosis of PNI is still poor.

PNIs may be caused by acute compression, laceration, or penetrating trauma resulting in the loss of sensory function, motor function or both. Nerve regeneration and recovery of nerve function depend on the type of nerve fiber injury, patient age, site of the lesion, length of the defect, level of damage of the surrounding tissues, and availability of neurotrophic factors.^4^ In the mildest kind of nerve injury, neurapraxia, the continuity of endoneurial tubes is preserved, and recovery occurs without Wallerian degeneration. However, neurotmesis and axonotmesis involve the loss of axonal continuity and the distal segment of injury undergoes Wallerian degeneration. Neurotmesis is the most severe type of nerve fiber injury including stretch injuries and laceration.^4^, ^5^ When the nerve gap is less than 5 mm, peripheral nerves can regenerate spontaneously with the support of Schwann cells (SCs), that promote a beneficial environment for axonal growth. As a response to denervation, SCs located in the distal axonal segment secrete a range of growth factors to facilitate regenerating axons to reach their sensory end organ or target muscle. In this case, end‐to‐end epineurial neurorrhaphy is suitable if tension free coaptation can be achieved after suturing the two stumps. For more severe injuries, implantation of an autologous nerve graft (autograft) is the gold standard procedure. However, nerve autograft may potentially involve further complications including scar tissue formation, donor site morbidity, lack of donor nerves and aberrant regeneration.^6^, ^7^ Therefore, new therapeutic strategies for peripheral nerve repair have focused on the development of nerve guidance conduits (NGCs) as alternatives to nerve autografts.

Current commercially available NGCs exhibit considerable drawbacks. For example, synthetic bioresorbable NGCs may produce an immune response, scar tissue, and release of by‐products that are detrimental for the regeneration process. Nonbiodegradable NGCs involve a second surgery for conduit removal, comprising an additional disadvantage.^8^ Hence, a diversity of materials, nanostructures and biochemical factors have been investigated in attempts to improve the performance of NGCs.^9^, ^10^, ^11^ Bioresorbable materials are preferred over non‐bioresorbable materials since they prevent both, chronic nerve compression and fibrotic reactions; and have been shown to produce a reduced risk of neuromas.^8^, ^12^ Although NGCs made from polymers of natural origin have shown a reduced immune reaction, the regeneration outcomes are not as good as the autograft when the gaps are longer than 10 mm.

Polyhydroxyalkanoates (PHAs), polymers of bacterial‐origin, are gaining increasing popularity, since they exhibit high biocompatibility, and tuneable biodegradability and mechanical properties.^13^ Studies have shown that D‐3‐hydroxybutyric acid (3HB), a natural constituent of blood,^14^ is a degradation product of some PHAs, which contributes to their high biocompatibility. Moreover, PHAs have shown superior biocompatibility with neuronal cells compared to the widely used synthetic polymers, polycaprolactone (PCL)^6^ and PLA.^15^, ^16^ PHAs exhibit properties that may overcome some of the limitations of the available NGCs including controllable surface erosion, lower acidity of their degradation products and longer‐lasting stability compared to their synthetic counterparts.

Although P(3HB) have previously displayed satisfactory nerve regeneration, its mechanical properties are unsuitable for peripheral nerve repair.^17^, ^18^ To overcome this limitation, we have fabricated NGCs by the dip molding technique using the biodegradable PHA‐blend 75:25 P(3HO)/P(3HB), which has been shown to possess the required flexibility and biocompatibility for this application.^16^ In the present study, we have carried out, for the first time, preclinical assessment of novel PHA blend‐based NGCs, PHA‐NGCs, for regeneration of median nerve gaps and functional repair by using the tubulation technique, with significantly promising results.

## 2 MATERIALS AND METHODS

### 2.1 Manufacturing of NGCs

PHA‐NGCs were fabricated from P(3HO)/P(3HB) 75/25 blend whose biocompatibility with neuronal cells was previously assessed by Lizarraga‐Valderrama et al.^6^ The NGCs were made by a multi‐dip molding process using a solution of P(3HO) and P(3HB) mixture (mass ratio 75 to 25) with a total polymer concentration of 6 wt% in chloroform. PTL‐MMB02 Programmable Dip Coater (MTI Corporation, Richmond, CA) was used for mandrel dipping into the polymer solution with a speed of 200 mm/min. NGCs were produced by using a six‐mandrel tool with mandrels of 1.8 mm outer diameter, along with a matching vial holder. Dip molding was conducted through five coating cycles consisting of five dips, resulting in a total of 25 dips. The drying time between dips in a coating cycle was 30 sec while the drying time between cycles was 4 min. After completing the coating cycles, the tubes were kept at room temperature for 3 days to complete solvent evaporation. Tubes were removed from the mandrels and stored for 5 weeks at room temperature (aged NGCs) before all the tests were carried out.

### 2.2 Characterization of the NGCs

#### 2.2.1 Scanning electron microscopy of PHA blend conduits

Surface topography of the PHA‐NGCs was analyzed using a FEI XL30 Field Emission Gun Scanning Electron Microscope (FEI, Netherlands). All the tubes were previously sputter‐coated with a 20 nm film of palladium using a Polaron E5000 sputter coater. The operating pressure of the sputter coating was 5 × 10^−5^ bar with a deposition current of 20 mA for a duration of 90 s. The images were then recorded at 5 kV using the FEI software.

#### 2.2.2 Mechanical analysis

Tensile testing was carried out using a 5942 Testing Systems (Instron, High Wycombe, UK) equipped with a 500N load cell at room temperature. NGCs of total length around 40 mm were fixed in rubber‐coated grips with the separation distance between the grips of 23 mm. Metal mandrels were inserted into the NGCs from both sides to the full gripping length (approximately 8 mm). Deformation rate was set to 10 mm per min. The average values for four specimens were calculated.

#### 2.2.3 Thermal analysis

Thermal transitions of NGC polymer blends were studied using DSC 214 Polyma (Netzsch, Germany), equipped with Intracooler IC70 cooling system. Scanning was conducted between −70 and 200°C at a heating rate of 10°C/min under the flow of nitrogen at 60 mL/min. Samples of known history (aged for 5 weeks at room temperature) were used in the DSC studies. Therefore, all thermal transitions were analyzed for the first heating which provided properties for the conditioned material. Enthalpy of fusion for each component of the polymer blend was normalized to a corresponding mass fraction.

### 2.3 In vitro proliferation and cell morphology analysis

RT4‐D6P2T cells were seeded at a density of 15,000 cells/cm^2^ on PHA NGCs and on glass cover slips used as controls and cultivated in Dulbecco's Modified Eagle Medium (DMEM, Sigma Aldrich) supplemented with 100 U/mL penicillin (Sigma), 0.1 mg/mL streptomycin (Sigma), 1 mM sodium pyruvate (Sigma), 4 mM L‐glutamine (Sigma) and 10% heat‐inactivated fetal bovine serum (FBS; all from Invitrogen). After 2 and 4 days in vitro (DIV), cells were fixed in 4% paraformaldehyde solution (PFA; Sigma‐Aldrich). Fixed cells were permeabilized with 0.1% Triton X‐100 for 1 h at room temperature. F‐actin was detected using TRITC‐conjugated phalloidin diluted 1:1000 in blocking solution (Chemicon‐Millipore) by 1 h incubation at room temperature followed by three wash steps of 5 min each and mounted with a Dako fluorescent mounting medium. Images were acquired using a Zeiss LSM800 confocal laser microscopy system (Zeiss, Jena, Germany). For each sample 30 images were taken, and the number of cells estimated using ImageJ software. For each acquired image, manual quantification was performed. The sections of the image where cells were not perfectly distinguishable from each other were excluded from quantification. As regards the analysis of cell morphology, 2 DIV‐time point was evaluated, for each image the area occupied by all the cells that were in focus was evaluated. To avoid contamination by the background, the area was manually traced using the appropriate plugin of the ImageJ software.

### 2.4 Surgery

For in vivo regeneration study, a total of 15 female adult Wistar rats were used (Harlan, weight: 200–250 g). Animals were housed in a room with controlled temperature and humidity, with 12 h of light and 12 h of dark, free access to food and water. Every attempt was made to reduce animal suffering. A preliminary pilot study was carried out on three female adult Wistar rats for qualitative analysis of nerve regeneration using PHA NGCs at 6 weeks from the injury. The other 12 animals were divided in two experimental groups (*n* = 6 PHA NGC group and *n* = 6 Autograft group as positive control) and sacrificed after 12 weeks. All procedures were approved by the Bioethical Committee of the University of Torino and by the Italian Ministry of Health. Moreover, these procedures agree with the National Institute of Health guidelines, the Italian Law for Care and Use of Experimental Animals (DL26/14), and the European Communities Council Directive (2010/63/EU).

**FIGURE 1:**
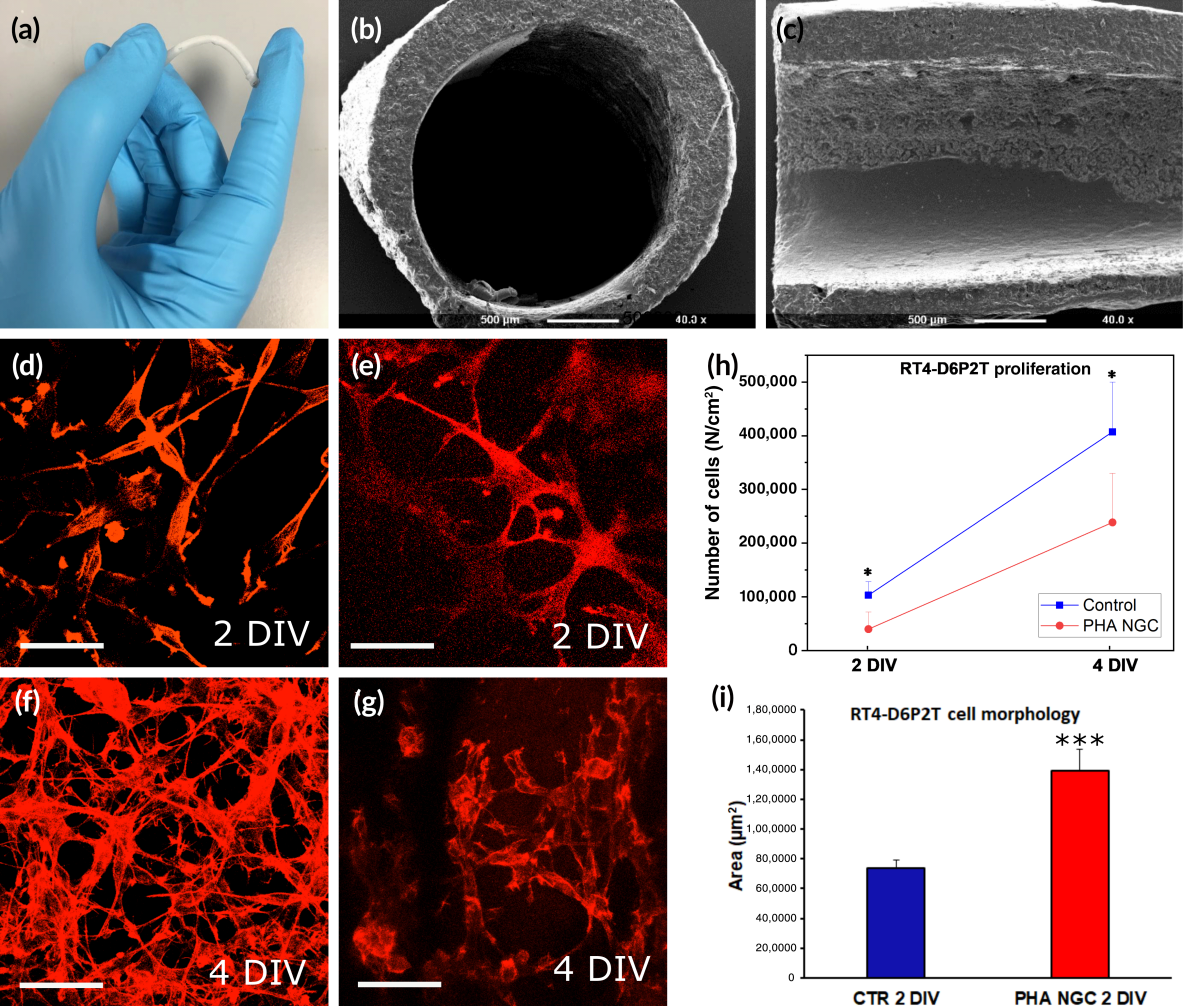
(a–c) Pictures and micrographs of the PHA‐NGC. (a) Photograph of the PHA‐NGC; (b) SEM micrographs of the cross section of the PHA‐NGC; (c) SEM micrographs of longitudinal section of the PHA‐NGC showing porous inner walls. D‐I: Results of in vitro analysis; (d‐g) Representative images depicting RT4‐D6P2T cells cultivated on the control substrate (d, f) and on the PHA‐NGC (e, g), stained with phalloidin. Scale bar: 20 μm; (h) Proliferation curve experiment. RT4‐D6P2T cell line were cultivated on the control substrate and on the luminal surface of the PHA‐NGC; (i) Cell morphology experiment: RT4‐D6P2T cell line were evaluated using the area covered after 2 days of culture *p < 0.05, ***p ≤ 0.001

During surgical procedures, animals were placed under general anesthesia induced by i.m. injection of Tiletamine + Zolazepam (Zoletil, 3 mg/kg) and were positioned in the supine position. Using an incision from the nipple to the elbow, the median nerve was isolated to establish a defect in the middle of the exposed part, immediately followed by nerve repair according to the experimental groups. In the experimental group PHA blend NGC, a median nerve segment was removed and 10 mm‐long conduits were inserted and sutured to the nerve stumps with one 9/0 epineural stitch to each nerve ends. In the Autograft group, the median nerve segments were removed, reversed (distal‐proximal) and sutured with three 9/0 epineural stitch to the nerve stumps of the same nerve (Figure 1).

PHA NGCs were immersed in sterile saline for at least 5 min before implantation. Animals were sacrificed by anesthetic overdose after 6 weeks for a qualitative observation of the ongoing regeneration (*n* = 3 PHA‐NGC) or after 12 weeks for quantitative analysis of nerve regeneration (*n* = 6 PHA‐NGC and *n* = 6 Autograft).

### 2.5 Functional evaluation of the regenerated nerve: The grasping test

The grasping test was performed to estimate the functional recovery after nerve reconstruction. The analysis was carried out every 2–3 weeks until the animal was sacrificed (12 weeks after surgery) following the same procedure previously described by Papalia et al.^19^ and Ronchi et al.^20^

### 2.6 Immunohistochemistry

The regenerated nerve inside the conduit (for the sample withdrawn after 6 weeks) was fixed in 4% paraformaldehyde for 2 h, washed in a solution of 0.01 M PBS (pH 7.2) for 30 min, dehydrated, and embedded in paraffin. Sections were cut 10 μm thick, permeabilized, blocked (0.1% Triton X‐100, 10% normal goat serum for 1 h) and incubated overnight with anti‐NF 200 kDa (monoclonal, mouse, Sigma Aldrich, dilution 1:200) and anti‐S100 (polyclonal, rabbit, Sigma Aldrich, dilution 1:300), at room temperature. Subsequently, sections were washed three times in PBS and incubated for 1 h at room temperature in a solution containing secondary antibodies: Alexa 488 anti‐Mouse (Molecular Probes, dilution 1:200), Cy3 anti‐Rabbit (Life Technologies, dilution 1:300). After three washes in PBS, sections were mounted with a Dako fluorescent mounting and analyzed using a Zeiss LSM800 confocal laser microscopy system (Zeiss, Jena, Germany).

### 2.7 Resin embedding, high resolution light microscopy and electron microscopy analysis

Regenerated nerve inside the conduit (withdrawn after 6 weeks) and regenerated nerves distal to the conduit/autograft (withdrawn after 12 weeks) were fixed by immediate immersion in 2.5% glutaraldehyde in 0.1 M phosphate buffer (pH 7.4) for 5–6 h at 4° C. Samples were then post‐fixed in 2% osmium tetroxide for 2 h and dehydrated in passages in ethanol from 30% to 100% (5 min each passage). After two passages of 7 min in propylene oxide and overnight in a 1:1 mixture of propylene oxide and Glauerts' mixture of resins, samples were embedded in Glauerts' mixture of resins (made of equal parts of Araldite M and the Araldite Harter, HY 964). 0.5% of dibutylphthalate was added to the resin mixture as a plasticizer. Finally, 2% of accelerator 964 (brand) was added to the resin in order to promote the polymerization of the embedding mixture, at 60°C.

Semi‐thin sections (2.5 μm thick) were cut using an Ultracut UCT ultramicrotome (Leica Microsystems, Wetzlar, Germany) and stained with 1.0% toluidine blue for high resolution light microscopy examination and design‐based stereology. A DM4000B microscope equipped with a DFC320 digital camera and an IM50 image manager system (Leica Microsystems, Wetzlar, Germany) was used for section analysis. With the same ultramicrotome, ultra‐thin sections (70 nm thick) were cut and stained with saturated aqueous solution of uranyl acetate and lead citrate. Sections were analyzed using a JEM‐1010 transmission electron microscope (JEOL, Tokyo, Japan) equipped with a Mega‐View‐III digital camera and a Soft‐Imaging‐System (SIS, Münster, Germany) for the computerized acquisition of the images.

### 2.8 Quantitative analysis of nerve regeneration: Stereological and morphometrical analysis

In order to quantify myelinated nerve fibers with high resolution light microscopy, one toluidine blue stained semi‐thin section was selected and the total cross‐sectional area of the whole nerve was measured. Thirteen to fifteen sampling fields were selected using a systematic random sampling protocol, as previously described by Geuna^21^ and Geuna et al.^22^ In each sampling field, a two‐dimensional dissector procedure was adopted to cope with the edge effect.^22^ Mean fiber density, total fiber number, fiber and axon diameter, myelin thickness and g‐ratio were then estimated.

### 2.9 Statistical methods

For statistical analysis IBM SPSS Statistics 22.0 software was used. Data were expressed as mean ± SD. Data were analyzed through the two‐tailed Student's *t*‐test. The level of significance was set at *p* ≤ 0.05 (*), *p* ≤ 0.01 (**), and *p* ≤ 0.001 (***).

## 3 RESULTS

### 3.1 Characterization of PHA‐NGCs

In this work, a dip‐coating technique was utilized for the fabrication of tubular NGCs. Dip molding is a highly flexible process which allows formation of polymer coatings from polymer solutions of a wide range of concentrations. Thickness of the coating depends on polymer concentration and viscosity of the polymer solution. Viscosity also is a significant parameter which influences the quality of the coating; the polymer solution must be fluid enough to allow leveling of imperfections before the transition of the coating into a non‐flowing semisolid layer. In a previous study,^6^ the 75:25 P(3HO)/P(3HB) blend showed appropriate mechanical properties for peripheral nerve regeneration and provided good support for neuronal cell attachment and growth. Hence, this polymer blend was chosen for the fabrication of NGCs presented in this study. The inner diameter, 1.8 mm, was close to that required for in vivo studies in rats (1.1–1.3 mm).

Our preliminary experiments demonstrated that due to the high molecular weight of the natural PHAs it was not possible to prepare processable solutions with the concentrations which would allow fabrication of tubes with sufficient wall thickness after a single dipping. Therefore, we adopted a multi‐dip molding process using a solution of 75:25 P(3HO)/P(3HB) mixture at a total polymer concentration of 6 wt%. Polymer solution of this concentration prevented an excessive downward flow (sagging effect) along a vertically fixed mandrel. A single dip into the polymer solution of this composition resulted in a 10 μm thick dry coating, whereas multiple dipping resulted in tubes with wall thickness of 200 μm. Chloroform, a highly volatile solvent used for polymer dissolution, was allowed to evaporate for a relatively short period of time, 30 seconds, before performing the next dipping. This short evaporation period led to a swollen, thick polymer layer on the mandrel and progressive growth of the thickness of the polymer coating. After every fifth coating, solvent evaporation was conducted for 4 min, before the start of the next five‐dip cycle. This prolonged evaporation period was required to induce further solvent losses by the gelled polymer material and stabilization of its thickness.

As can be seen from Figure 1, semitransparent tubes (a) were produced using this optimized procedure. A slightly irregular wall thickness was observed by SEM imaging of sectioned samples (b, c). Both longitudinal and transverse cross‐sections confirmed the structural integrity of the PHA blend without the signs of layered structure formation or inter layer delamination. These observations demonstrated the robustness of the optimized multi‐dip molding process in the fabrication of PHA‐based tubes. It must be noted that a dispersed phase is discernible in the morphological structure exposed in the cross‐sections. This suggests the formation of an immiscible PHA blend (SI, Figure S1). However, the gaps between the inclusions of the dispersed phase and continuous phase are not evident which implies good adhesion between the phases.

The NGCs were characterized by a high flexibility inherited from the elastomeric P(3HO) (SI, Figure S1). These NGCs could withstand deformations up to 150% which was larger than rat sciatic nerves can resist. Ultimate tensile strength and Young's modulus of the aged NGCs were found to be 6 and 35 MPa, respectively (Table 1). The Young's modulus of the NGC is 60 times higher than rat sciatic nerve (Table 1). Although the stiffness of NGCs is significantly higher compared to that of the rat sciatic nerves, it should be taken into consideration that the NGCs were tested in a dry state and the stiffness is expected to decrease for the wet NGCs after implantation. However, these changes cannot be significant since PHAs, particularly P(3HO), are hydrophobic polymers and their swelling in aqueous media is limited. Also, higher stiffness of NGCs can be beneficial to prevent the collapse of the hollow structure after implantation.

**TABLE 1:** Mechanical properties of NGCs

	Ultimate tensile strength, MPa	Elongation at break, %	Young's modulus, MPa
PHA NGC	6.0 ± 1.0	130.0 ± 20.0	35.0 ± 3.0
Rat sciatic nerves^a^	2 0.7 ± 0.9	NA	0.58 ± 0.2

^a^ Borschel et al.^23^

### 3.2 In vitro proliferation and cell morphology assay

In vitro proliferation and cell morphology assays, using the RT4‐D6P2T cell line, were performed to evaluate the ability of the glial cells tested to make direct contact with the substrate represented by PHA‐NGC and consequently to determine the biocompatibility and the biomimetic properties. RT4‐D6P2T proliferation was significantly higher on the control compared to PHA‐NGCs (Figure 1h). However, RT4‐D6P2T morphology at 2 DIV was very well organized and cell dimensions significantly higher on the PHA‐NGCs compared to the control (Figure 1i).

### 3.3 Surgical procedure

**FIGURE 2:**
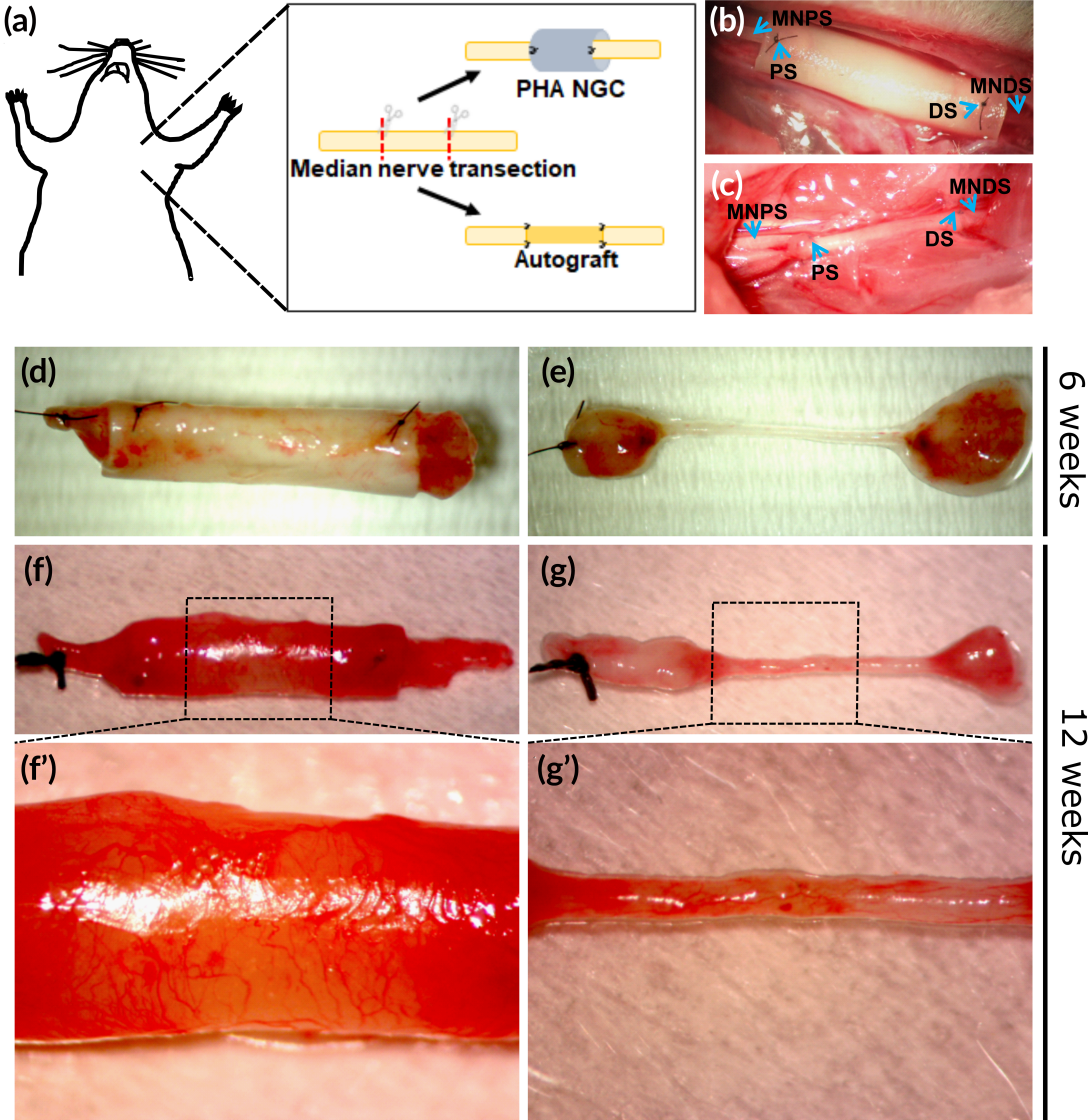
(a‐c) Experimental design and photographs of the surgery. (a) Experimental design of transection and repair of rat median nerve. The median nerves were transected and immediately repaired with PHA‐NGCs (b) or nerve autograft (c). MNPS: Median nerve proximal stump; MNDS: Median nerve distal stump; PS: Proximal suture; DS: Distal suture. (d–g) Photographs of PHA‐NGC removal. Regenerated nerves were obtained from rats sacrificed at 6 weeks (d, e) and from rats sacrificed at week 12 (f, g) post‐surgery. A magnification showing the new vascularization is also shown (f′, g′). After withdrawal, conduits were removed, and the regenerated nerves were processed for analysis

The rat median nerve repair model was used as a preclinical test model to evaluate the performance of the PHA‐NGCs in promoting peripheral nerve regeneration in vivo (Figure 2a). The PHA‐NGCs were implanted in injured median nerves to repair 10 mm long defects (Figure 2b) and compared to the “gold standard” technique, nerve autograft (Figure 2c). During the surgical procedures the handling and suturability of PHA‐NGCs were evaluated qualitatively; they proved to be flexible, easily implantable and easy to suture, demonstrating an adequate tear‐resistance feature. Due to the mechanical properties of the material, the stiches did not negatively affect the structural integrity of PHA‐NGCs. Although PHA‐NGCs were semi‐transparent, the nerve stumps were readily located under a light microscope allowing a verification that the nerve transection was precise. All rats were in good health condition throughout the experiment. No sign of inflammation, pain, discomfort or apathy were reported. Moreover, no sign of auto‐mutilation, ulcers or joint contractures were observed.

### 3.4 Gross examination of PHA‐NGCs after 6 and 12 weeks

During explantation, macroscopic analysis of the PHA‐NGCs was qualitatively conducted at 6 and 12 weeks after implantation (Figure 2d–g). Following animal sacrifice, gross inspection of the surgical sites revealed that all the devices were still clearly recognizable and were found on site with both nerve ends still connected. The external structure of the PHA‐NGCs remained stable and was covered with a thin layer of connective tissue, demonstrating remarkable toleration by the host tissues without causing a detectable immune response (Figure 2d,f). No signs of inflammation or scar tissue formation around the PHA‐NGC was observed, showing optimal biocompatibility with the peripheral nerve tissues. Moreover, a large number of blood vessels were newly formed around the PHA‐NGC (Figure 2f,f′), and inside the regenerated nerve (Figure 2g), as shown at higher magnification in Figure 2f′,g′. Finally, at each time point the PHA‐NGC were not degraded, neither were they embrittled, had a semi‐transparent appearance and proved to be soft and flexible.

### 3.5 Results of the pilot in vivo study: qualitative analysis of nerve regeneration inside PHA NGC after 6 weeks

**FIGURE 3:**
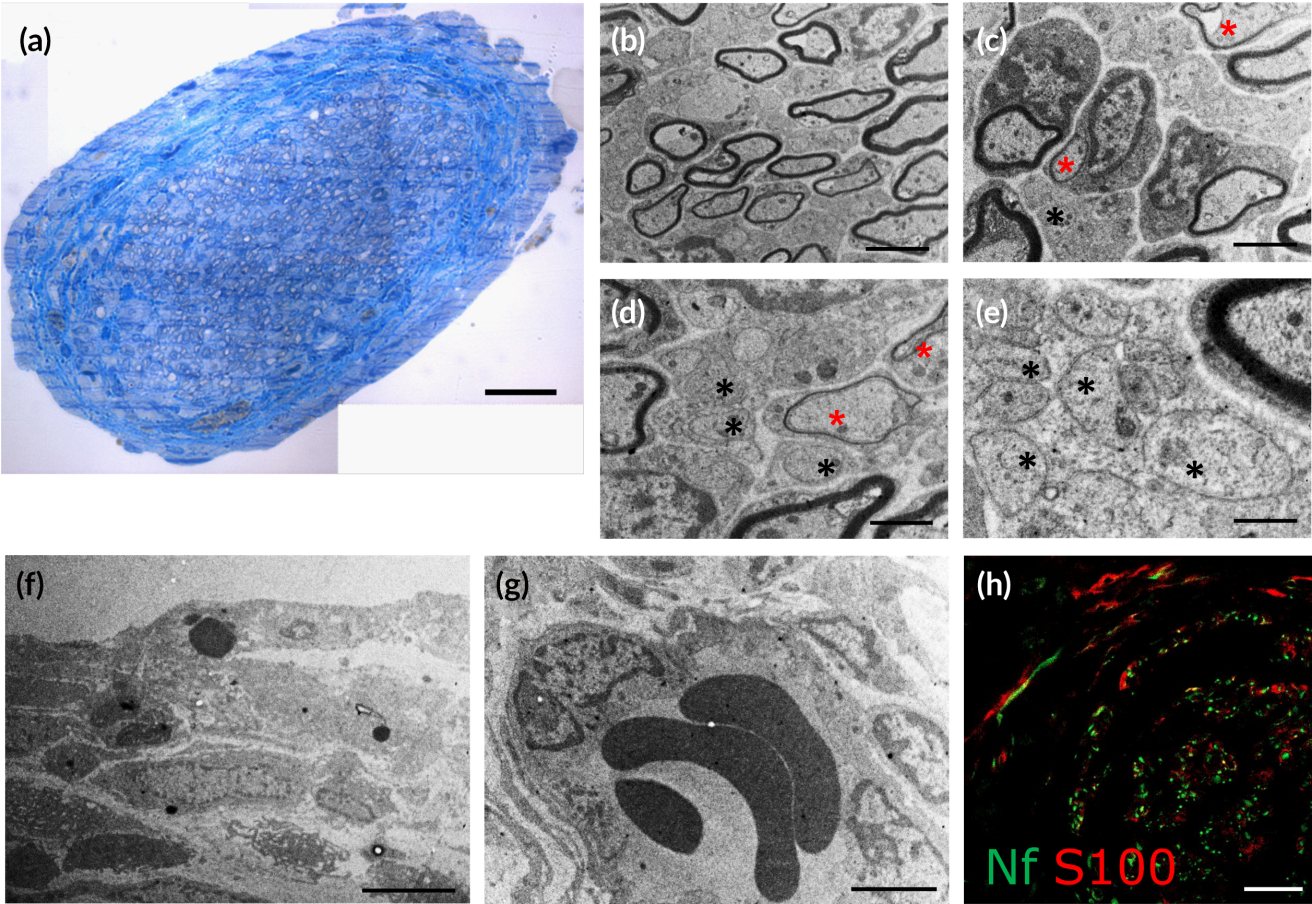
(a‐h) Qualitative morphological examination of regenerated median nerve after 6 weeks. (a) Toluidine blue–stained semi‐thin cross section of the regenerated nerve in the central portion of the PHA‐NGC showed several regrowing fibers surrounded by a layer of connective tissue. (b–e) Electron microscopy micrographs showing both, myelinated (easily visible due to a thick myelin sheath) and unmyelinated (shown with black asterisks) fibers. Axons surrounded by a thin layer of myelin are shown with a red asterisk. (f) Connective tissue surrounding the regenerated nerve. (g) Detailed view of newly formed blood vessels. (h) Immunohistochemical staining of neurofilament (green) and S100 protein (red) revealed the association between axons and Schwann cells. Scale bars: a, h: 20 μm; b, c, g: 2 μm; d: 1 μm; e: 0.5 μm; f: 5 μm

After removal of the PHA‐NGCs, axonal regeneration was assessed in the central portion of the regenerated nerves by toluidine‐blue staining and immunohistochemistry. Toluidine blue‐stained semi‐thin cross section of the regenerated nerve in the central portion showed several regrowing fibers surrounded by connective tissue (Figure 3a). The electron micrographs presented in Figure 3 showed both, myelinated (Figure 3b,c) and unmyelinated regrowing fibers (Figure 3d,e) in association with Schwann cells. Several regrowing fibers with thin myelin sheath were observed (Figure 3d,e), demonstrating that the myelination and maturation processes were in place at week 6. The epineurium was clearly observable and well‐formed, characterized by rich axonal growth (Figure 3a). Schwann cells surrounding axons with sheaths of myelin were clearly observed. Axons and Schwann cells were also immunolabeled for neurofilament and S100 protein, respectively. Figure 3h shows the distribution of nerve fibers (green) and Schwann cells (red) within the nerve cross‐section.

### 3.6 Functional recovery

**FIGURE 4:**
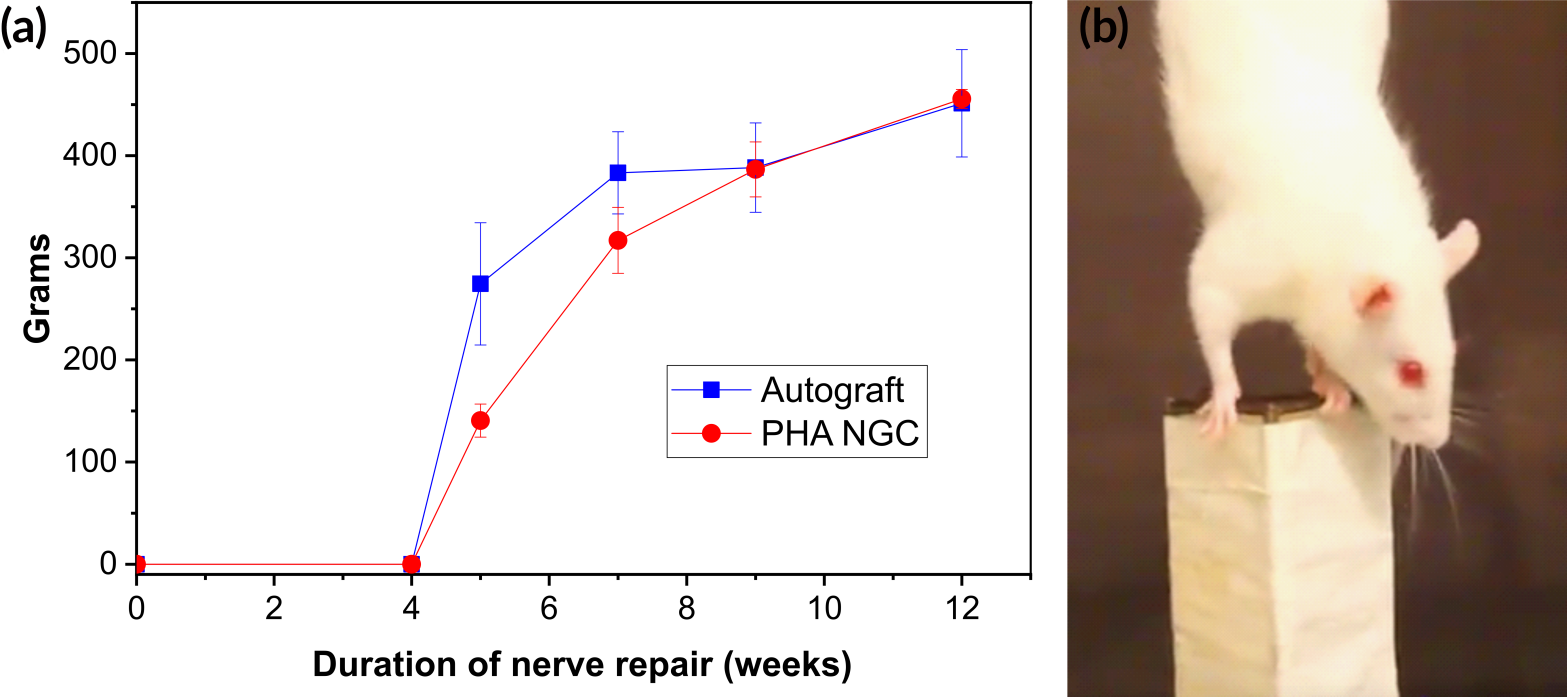
(a) Results of the functional recovery of the median nerve. Posttraumatic time course of the grasping test revealed an increased performance of functional recovery in the autograft group compared with the PHA‐NGC group at week 5 post‐surgery. At week 7, the two experimental groups showed similar functional recovery values until week 12. Values are presented as mean ± SD, *p ≤ 0.05. (b) Photograph showing a rat grasping the bar of the device with the operated paw (left limb)

**FIGURE 5:**
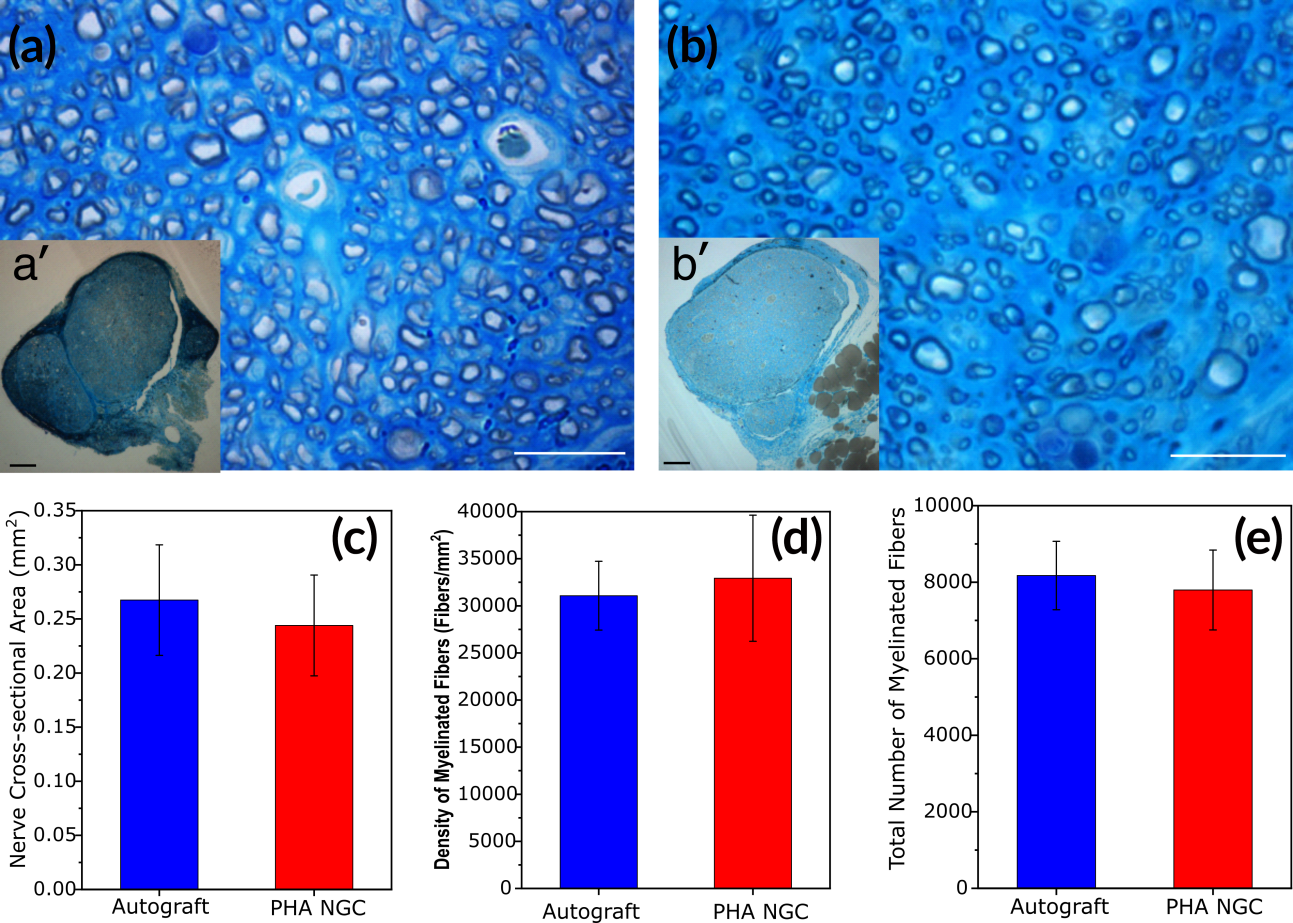
(a and b) Representative high and low magnification light photomicrographs of toluidine blue–stained semi‐thin cross sections of the distal stump of the regenerated nerve repaired with autograft (a and a′) or PHA NGC (b and b′) after 12 weeks. Scale bars: a, b: 20 μm; a′, b′: 100 μm. c, e: Results of the stereological analysis; (c) cross sectional area of the regenerated nerves; (d) density of regenerated myelinated fibers; (e) total number of regenerated myelinated fibers. No significant differences between the two groups were found. Values are presented as mean ± SD, *p ≤ 0.05

To assess the functional recovery of the median nerve, the grasping test was performed in all operated animals at week 2, 4, 5, 7, 9, and 12 post‐surgery (Figure 4). At week 2 and 4, the function of finger flexor muscles innervated by the median nerve was 0 g in both, autograft and PHA‐NGC groups, confirming a complete nerve fiber transection. The function of the muscles started to recover faster in the autograft group, reaching a performance statistically different compared with the PHA‐NGC group at week 5. The two groups reached comparable values at week 7, showing no statistical differences until week 12 post‐surgery (Figure 5e).

### 3.7 Morphological, morpho‐quantitative, and stereological analysis of regenerated median nerve after 12 weeks

Morphological evaluation at higher magnification revealed that both PHA‐NCG and nerve autograft (Figure 5a,b) exhibited several regrowing myelinated fibers with a well‐defined axoplasm and well‐organized myelin sheath. Design‐based stereological analysis and morpho‐quantitative analysis were performed using one randomly selected toluidine blue stained semi‐thin section cut distally to the PHA‐NGC (Figure 5). Semi‐thin cross sections of the distal stump of the regenerated nerves from both groups, autograft (Figure 5a) and PHA‐NGC (Figure 5b) after 12 weeks repair were examined. The nerve cross sectional area for both groups was found to be similar after 12 week's repair (Figure 5c).

The density of the myelinated fibers resulted in similar outcomes for both groups, autograft and PHA‐NGCs, without showing any significant difference (Figure 5d). Similarly, the difference between the total number of myelinated fibers for the two groups was not significant (Figure 5e).

**FIGURE 6:**
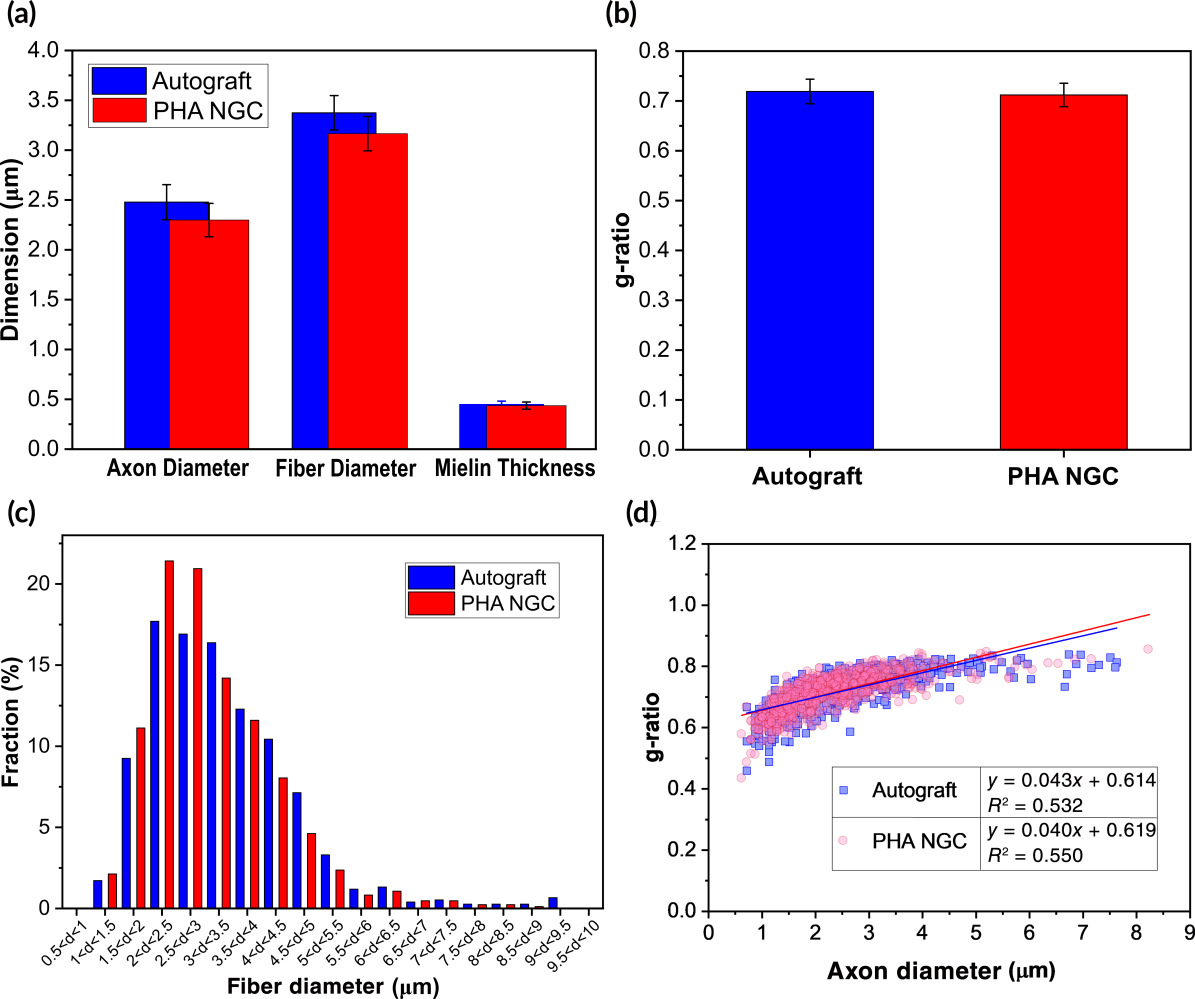
(a–d) Results of the morphometrical evaluation of regenerated nerve fibers. (a) Morphometrical evaluation of size parameters: axon diameter, fiber diameter and myelin thickness showed no significant differences between the two groups. (b) The g‐ratio was similar for both groups. (c) The frequency distributions of fiber diameters showed a higher percentage of smaller fibers in the PHA‐NGC group. (d) The g‐ratio/axon diameter correlation of individual fibers showed that the resulting linear regression lines of the autograft and the PHA‐NGC group were similar. PHA‐NGC: 845 fibers analyzed; Autograft: 757 fibers analyzed. Values are presented as mean ± SD, *p ≤ 0.05

Morpho‐quantitative measurement of size parameters was also found similar for both groups (Figure 6a). No significant differences were found between axon diameter, fiber diameter and myelin thickness displayed for the autograft and the PHA‐NGC groups (Figure 6a). However, the frequency distributions of nerve fiber diameters showed a higher percentage of fibers with a smaller diameter in the PHA‐NGC group compared to the autograft group (Figure 6c). In the PHA‐NGC group 81.4% of myelinated fibers showed a diameter smaller than 4 μm, whereas in the autograft group the percentage was 74.2%. The parameter g‐ratio, calculated as axonal diameter to the total outer diameter is one of the more reliable morphological predictors of nerve recovery (Figure 6b,d). As shown in Figure 6b, the g‐ratio was also found to be remarkably similar for both groups. Moreover, the g‐ratio/axon diameter correlation of individual fibers confirmed similar regeneration outcomes (Figure 6d). Finally, the linear regression lines of autograft and PHA‐NGC group overlapped as shown in Figure 6d.

## 4 DISCUSSION

Peripheral nerves are characterized by a remarkable ability to regenerate following a transection injury, consisting of a complex and poorly understood multicellular response. However, this process might be considerably hindered when injury gaps are longer than 5 mm.^8^ The natural regenerative process in peripheral nerves involve distinct stages lead by various cells including neurons, SCs, macrophages, fibroblasts, endothelial cells, and neutrophils.^24^, ^25^ Following the severing of the nerve, the stumps retract and secrete plasma exudate rich in neurotrophic factors and ECM precursor molecules including fibrinogen and factor XIII.^25^, ^26^ A “bridge” composed of perineurial cells, inflammatory cells, fibroblasts and matrix is formed to reconnect the two nerve stumps.^25^ When regeneration occurs within a hollow tube, initially an acellular fibrin cable forms between the two stumps, followed by migration of SCs, endothelial cells and fibroblasts along the cable.^26^ This fibrin cable normally forms within 1 week across a noncritical 10 mm gap in rat models.^26^, ^27^ After cellular migration, degradation and removal of the fibrin cable lead to the onset of remyelination by switching from progenitor‐like phenotype SCs to a more mature “myelinating” phenotype. As a result, these mature SCs wrap around the regenerated axons to form the myelin sheath.^26^ The regenerative process inside the PHA‐NGCs followed the same phases, with the formation of a thin regenerated nerve inside the PHA‐NGCs after 6 weeks and still a thin (but bigger as compared to 6 weeks) regenerated nerve after 12 weeks. In both time points the cross section of the regenerated nerve appeared to be thinner as compared to both the diameter of the conduit and the diameter of the nerve stumps, but increased over time. Also, in both time points the regenerated nerve grew in the middle of the conduit. In our experience, this is a normal process in nerve regeneration: regenerated nerve fibers start to grow where the fibrin cable was located and appear to have a thin cross‐section, at least for the first weeks/months. We cannot exclude the fact that the space between the regenerating fibers and the inner wall of the PHA‐NGCs was colonized by some material (in particular ECM), but unfortunately, after the removal of the conduit for subsequent analysis, this material was lost.

Indeed, to perform quantitative stereological and morphometrical analyses on resin‐embedded nerves, we removed the PHA‐NGCs before the fixation with glutaraldehyde. Resin embedding and toluidine blue staining of nerve cross section allowed to perform a reproducible and standardized assessment of the degree of nerve regeneration, by preserving the fine structure of the nerve tissue and by providing high quality and clear detailed images of nerve fibers (and in particular of the myelin sheath).^28^ On the other hand, by removing the PHA‐NGCs, we were not able to investigate the direct interaction between the regenerated nerve and the wall of the conduit. We will endeavor to carry this out in future studies.

In vitro tests performed on the PHA‐NGCs depicted that the glial RT4‐D6P2T cells showed a very well‐organized morphology and proliferated efficiently on this regenerative substrate. In particular, the phalloidin staining allowed to highlight a marked organization of the actin filaments and an increase in the area occupied by the cells. These data are in good agreement with previous studies, where the in vitro biocompatibility in terms of adhesion, proliferation and differentiation on electrospun P(3HB)/P(3HB‐co‐3HV) nanofibers was tested with SCs.^29^

High‐resolution light microscopy allowed the visualization of myelinated fibers surrounded by connective tissue. A rich bunch of small myelinated nerve fibers was detected after 6 weeks of repair, indicating that robust axonal regeneration occurred inside the PHA‐NGCs. Moreover, axons and Schwann cells were clearly visualized by immunolabeling of neurofilament and S100, respectively. This analysis showed the association of nerve fibers with Schwann cells, confirming that nerve repair occurred normally. Also, blood vessels and connective tissue formation were observed during nerve repair using the PHA‐NGCs without showing formation of scar tissue. Hence, the absence of scar tissue and the presence of newly formed blood vessels have clearly shown that the PHA‐NGCs not only have the ability to support the intrinsic regenerative process of peripheral nerves but also exhibit high level of biocompatibility. Hence, in summary, the results of the pilot study carried out after 6 weeks confirmed the ability of PHA‐NGCs to support peripheral nerve repair.

For longer time points (12 weeks post‐surgery), morphometric analysis was performed to assess parameters of the nerve fiber population including axon diameter, fiber diameter, myelin thickness, and g‐ratio. Nerve morphology provides essential information related to functional recovery. As stated previously, axon and fiber diameter and myelin thickness were similar in both groups after 12 weeks post‐surgery. These parameters are vital for the study of the regeneration outcome since they affect the conduction velocity of the nerve impulse.^30^ During axonal maturation, an increase in axon and fiber diameters and internodal distance results in an increase in motor nerve conduction velocity (mNCV).^31^ Therefore, similar values presented in axon and fiber diameter and in myelin thickness in both groups could explain the similar level of functional recovery found in the grasping test results for both groups. On the other hand, the fiber diameter distribution was found to be different between the groups. Regenerated nerves from the PHA‐NGC group showed a higher percentage of fibers with smaller diameters as opposed to the autograft group. Nevertheless, this difference did not seem to have any impact in the functional recovery. g‐ratio, a parameter widely used as a functional and structural index to evaluate axonal myelination,^32^ was found to be similar in both groups. Hence, the myelination levels presented by both experimental groups were similar at week 12 of nerve repair.

Functional recovery of regenerated nerve after surgery was accessed by the grasping test during a period of 3 months. This behavioral method allowed the qualitative and quantitative evaluation of the flexor function in rat median nerve. Grasping response is a complex sensory‐motor response integrating sensory afferents with motor afferents through the cerebral cortex.^33^ Flexion of the fingers depends on two nerves, the median and the tibial nerves located in the forelimb and in the hindlimb, respectively.^33^ A common feature between humans and rats is the possession of prehensile forelimbs, forepaws and digits resulting in remarkable similarities between the two species.^34^ These striking resemblances makes the grasping test a powerful translational tool.^35^ However, the sciatic nerve injury model is currently preferred over the median nerve injury model mainly because of the large size of the sciatic nerve, which facilitates animal surgery. Furthermore, majority of the published studies in peripheral nerve regeneration use this model allowing comparative analysis. This makes the median nerve model's results difficult to compare with the current literature. Nevertheless, experimental results obtained using the grasping test are more likely to be translated to clinical trials since the PNI is higher in upper extremity nerves compared to nerves located in the lower limb. Furthermore, the majority of the nerve repairing surgical interventions involve radial, median, ulnar and auxiliary nerves. Moreover, the use of the median nerve model has demonstrated an increased preserved animal welfare following nerve transection compared to the sciatic injury model.^19^, ^36^ In the present study, the grasping test allowed the comparative evaluation of the median nerve function post‐surgery in the two groups under study. Although the autograft group exhibited better performance at grasping, at week 5, compared with the PHA‐NGC group, comparable performance was observed in the two groups from week 7 until the end of the experiment. The delay of functional recovery of the PHA‐NGC group compared to the autograft group is justified by the different repair technique. Hence, PHA‐NGCs have demonstrated optimal performance in supporting not only nerve regeneration but also functional recovery.

The only PHAs that have been used and reported as base material for the manufacturing of NGCs are P(3HB),^17^, ^18^ poly(3‐hydroxybutyrate‐co‐3‐hydroxyvalerate) (P(3HB‐co‐3HV),^37^ and poly(3‐hydroxybutyrate‐co‐3‐hydroxyhexanoate) (P(3HB‐co‐3HHx)).^38^ Despite the fact that P(3HB) has displayed high biocompatibility with neuronal cells in all these studies, this polymer lacks suitable mechanical properties for peripheral nerve regeneration. In fact, P(3HB) is a well‐known brittle polymer widely used in bone tissue engineering with a Young's modulus and a tensile strength of 1200 ± 200 and 30 ± 2 MPa, respectively.

Mechanical compliance of neural implants and scaffolds with their target tissues is fundamental not only for allowing a greater freedom of movement in the affected area but also to produce a favorable environment for optimal regeneration. Although the Young's modulus and tensile strength of the PHA‐NGCs (35 ± 3 and 6 ± 1 MPa) (Table 1) were higher than the respective values of peripheral nerves in rats (0.58 ± 0.16 MPa and 1.4 ± 0.29 MPa), the regeneration displayed was optimal and similar to an autograft. There is also substantial evidence showing that the behavior of neuronal and glial cells are affected by the surrounding mechanical environment.^39^ Balgude et al.,^40^ subjected DRG neurons to a range of mechanical environments with agarose. The rate of the neurite extension was found inversely correlated to the mechanical stiffness of agarose gels. Moreover, Willits and Skornia,^41^ studied the effects of varying mechanical properties of collagen gels in neurite extension using chick DRG. They found maximum neurite extension in lower concentration gels. Hence, the mechanical properties of the manufactured PHA‐NGCs seem to have a favorable influence on neurite extension. Most of the commercially available NGCs do not possess the flexibility required to match the mechanical properties of neural tissue,^42^ which could hamper efficient neurite extension leading to a poor recovery of the nerve function, while limiting movement of the affected area. For example, the Young's modulus of Neurotube®, NeuraGen®, and Neurolac® are 4.00, 0.08, and 0.14 MPa, respectively,^43^, ^44^ differing from 0.58 MPa^23^ presented in fresh rat sciatic nerves. Moreover, Neurotube®, with a tensile strength of 13 ± 3 MPa, lacks the adequate strength to support peripheral nerve regeneration. Tensile strength of the median nerve in humans has been reported between 35.01 and 53.25 MPa.^45^

Although dip‐molding technique was effective for the manufacturing of conduits to perform in vivo studies, other techniques can be used in order to manufacture NGCs with more regular diameters such as injection molding or melt extrusion.^46^, ^47^ The latter will perhaps be the most scalable manufacturing method to be used for future commercialization of the device.

## 5 CONCLUSIONS

The results of the present study demonstrate, for the first time, that NGCs made using the bioresorbable PHA‐blend 75:25 P(3HO)/P(3HB) can successfully sustain cell proliferation and adhesion in vitro and nerve regeneration across a 10 mm median nerve defect in vivo. The conduit has proven to be biocompatible with the surrounding tissue, since no signs of inflammation or scar tissue formation were found. Also, our PHA‐NGCs, with a diameter of 1.8 mm is suitable for human nerve size, especially for digital nerve repairs, which are the most frequently severed peripheral nerves. Moreover, this hollow NGC could provide an excellent scaffold to design and develop engineered nerve grafts used to repair longer nerve gaps in the future, together with gene/cell therapy approaches. Further investigation of this NGC will focus on optimization of the conduit structure and properties, including wall permeability and biodegradation in vivo.

## CONFLICT OF INTEREST

The authors declare no conflict of interest.

## AUTHOR CONTRIBUTIONS

**Lorena del Rosario Lizarraga Valderrama:** Conceptualization; data curation; formal analysis; investigation; methodology; project administration; validation; visualization; writing‐original draft; writing‐review & editing. **Giulia Ronchi:** Conceptualization; data curation; formal analysis; investigation; methodology; project administration; validation; visualization; writing‐original draft; writing‐review & editing. **Rinat Nigmatullin:** Conceptualization; data curation; investigation; methodology; writing‐original draft. **Federica Fregnan:** Investigation; writing‐review & editing. **Pooja Basnett:** Investigation; writing‐review & editing. **Alexandra Paxinou:** Investigation; writing‐review & editing. **Stefano Geuna:** Conceptualization; funding acquisition; project administration; resources; supervision; writing‐review & editing. **Ipsita Roy:** Conceptualization; funding acquisition; project administration; resources; supervision; writing‐review & editing.

### PEER REVIEW

The peer review history for this article is available at https://publons.com/publon/10.1002/btm2.10223.

## Supporting information

**Appendix S1**: Supplementary Information

## Data Availability

The data that support the findings of this study are available from the corresponding author upon reasonable request.
