# Supplementary material for: Preclinical study of peripheral nerve regeneration using nerve guidance conduits based on polyhydroxyalkanaotes
Source: Bioeng Transl Med. 2021 May 21;6(3):e10223. doi: 10.1002/btm2.10223 (PMC8459605; doi:10.1002/btm2.10223)
Supplement: Supplementary file 1 — Appendix S1: Supplementary Information [file BTM2-6-e10223-s001.docx]

**Supporting information**


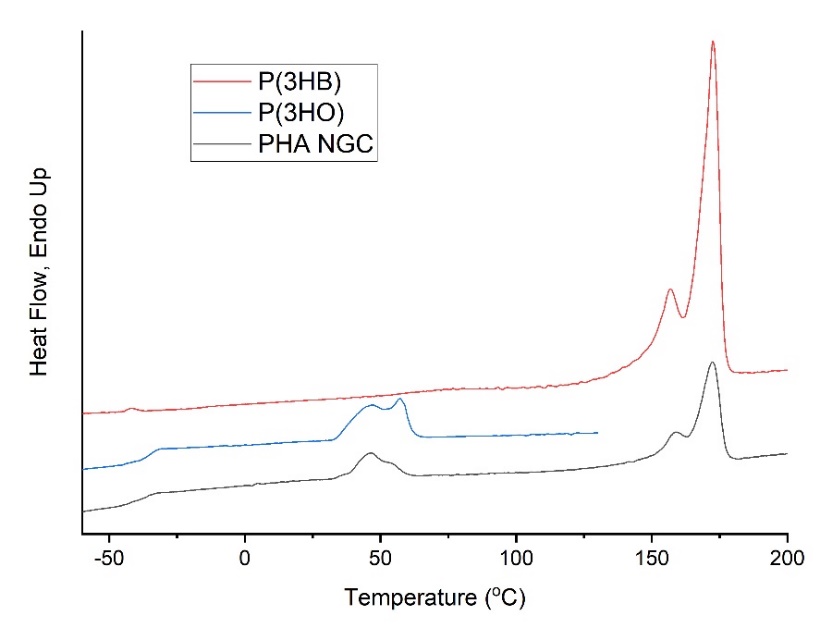


**Thermal properties of NGC and blend components**

|  | **P(3HO)** | | | **P(3HB)** | | |
| --- | --- | --- | --- | --- | --- | --- |
|  | T_g1_ | ΔH_1_, normalised with respect to P(3HO) | T_m1_ | T_g1_ | ΔH_1_, normalised with respect to P(3HB) | T_m1_ |
| **PHA NGC** | -37.5 | 10.31 | 56.3 | n/d | 99.84 | 177.3 |
| **P(3HO)** | -37.6 | 16.73 | 61.6 | - | - | - |
| **P(3HB)** | - | - | - | n/d | 85.9 | 176.6 |

**Figure S1**. DSC heating thermograms of P(3HB), P(3HO) and 75:25 Poly(3-hydroxyoctanoate)/P(3-hydroxybutyrate) blend (PHA NGC) and thermal properties of NGCs. DSC thermograms of the first heating demonstrated thermal events characteristic for the individual components of the polymer blend. No shifts in the position of P(3HO) glass transition temperature and temperature range of melting of both P(3HO) and P(3HB) were observed for the NGC in comparison to the corresponding thermal events of pure components. The enthalpy of melting increased for P(3HB) but decreased for P(3HO) in comparison with the corresponding phase transition observed for pure PHAs aged under similar conditions. P(3HB) crystallized to a higher degree in a matrix of soft P(3HO). Crystallization of P(3HO) was suppressed in the presence of P(3HB). In this PHA blend, the dispersed phase of rigid P(3HB) contains an elevated crystalline fraction while the amorphous fraction of soft P(3HO) increased in the continuous phase, providing a tougher material.
